# Effect of phenolic anchor groups on enzymatic polymerization of coniferyl alcohol at cellulosic interfaces

**DOI:** 10.1038/s41598-025-18530-9

**Published:** 2025-09-12

**Authors:** Thomas Elschner, Jakob Schönrich, Matej Bračič, Tina Maver, Uroš Maver, Steffen Fischer

**Affiliations:** 1 Institute of Plant and Wood Chemistry, Dresden University of Technology, Pienner Str. 19, 01737 Tharandt, Saxony Germany; 2 Faculty of Mechanical Engineering, Institute of Engineering Materials and Design, University of Maribor, Smetanova 17, 2000 Maribor, Slovenia; 3 Department of Pharmacology, Faculty of Medicine, University of Maribor, Taborska Ulica 8, 2000 Maribor, Slovenia

**Keywords:** Dehydrogenation polymerization, Artificial lignin, Phenolic cellulose esters, Thin films, QCM-D, Biochemistry, Chemistry, Materials science

## Abstract

The chemical recalcitrance of lignin limits the industrial processing of biomass, which could be addressed by so-called designer lignins. Dehydrogenation polymers (DHPs) formed by artificial lignification of monolignols, enable studies on structure-property relationships independently of genetic information. Thin films of phenolic acid esters of cellulose were prepared and used for quartz crystal microbalance with dissipation monitoring (QCM-D) experiments to investigate surface polymerization in real-time. The phenolic anchor groups significantly influenced lignification speed, deposited mass, and rigidity of resulting DHP layers. Linkage types in the lignin structure were quantified by HSQC NMR spectroscopy. Polymerization efficiency was increased in the order ferulate < p-coumarate < caffeate. Among the tested anchors, protocatechuate groups were excellently performing the reaction, while vanillate and p-hydroxybenzoate led to minimal deposition of DHPs. Lignification behavior could be correlated with radical stability of phenolic anchor groups and the formation of benzodioxane structures of caffeate moieties. The presence of caffeate units that undergo trapping reaction, prevents cross-linking of cell wall components and enhances digestibility. Moreover, the benzodioxane motif increased rigidity and linearity of lignin, which is advantageous for material science applications, e.g. for bio-based carbon fibers.

## Introduction

Lignin is a complex phenolic macromolecule that is abundant in secondary plant cell walls and allows the compressive strength of plant tissue, water transport, and defense against pathogens^1^. However, the chemical recalcitrance of lignin is the key obstacle for the industrial processing of biomass in the fields of pulping^2^, biofuels^3^, and agriculture^4^. Therefore, a series of research attempts to adjust lignin in biomass have been performed. Genetic, computational, and chemical approaches can be distinguished.

First, genetic engineering has been applied to reduce lignin content, which unfortunately results in diseased plants^5^. However, lignin could be engineered for improved utilization of biomass. The so-called designer lignins are aimed at a low degree of polymerization, less hydrophobicity, less structural cross-linking to carbohydrates, and chemically labile bonds. Moreover, tailoring of functionalities for high-value products by using lignin waste streams is an issue^6^.

Second, computer simulations allow prediction of lignin biosynthesis^7^, formation from monolignol radicals^8,9^, and molecular interactions between lignin and hemicellulose^10^.

In the context of our work, the artificial polymerization of monolignols to yield dehydrogenation polymers (DHPs) enabling hypotheses about structure-property relationships without considering genetic information is the third approach. Since Freudenberg performed dehydrogenative polymerization in vitro for the first time^11^, many researchers attempt on the oxidative coupling of sinapyl-, coniferyl-, and coumaryl alcohol by hydrogen peroxide in the presence of peroxidases. For example, the influence of pH value, scale up, addition of organic solvent, and differences between Zutropf (ZT) and Zulauf (ZL) modes on the linkage type and molecular weight were investigated in a recent study^12^. The reactions were systematically carried out from homogeneous buffer solutions to control the chemical structure. Another promising approach is the utilization of planar interfaces to model the cell wall environment, i.e. the formation of synthetic lignin on, e.g., hemicellulose films^13,14^.

Owing to their well-defined properties, cellulosic thin films are excellently suited for adsorption studies^15^ and chemical surface modifications^16,17^. Spin-coating and the Langmuir-Blodgett technique of trimethylsilyl (TMS) cellulose may yield very smooth films after the cleavage of TMS groups applying hydrochloric acid vapor^18,19^. These films can be applied in combination with quartz crystal microbalance with dissipation monitoring (QCM-D), which is a powerful analytical tool enabling real-time monitoring of surface phenomena. The measuring principle exploits the change in the oscillating frequency of a piezoelectric quartz crystal upon mass loading and allows the detection of the mass adsorbed on a surface^20–23^. Online measurements of frequency shifts ($\Delta$F) and dissipation factor changes ($\Delta$D) allow conclusions about the mass and viscoelastic properties of the layers to be drawn^24^. The Sauerbrey equation^25^ describes the proportionality of the adsorbed mass to the change in frequency, which is valid for rigid films ($\Delta$F/$\Delta$D >25)^26^.

The deposition of DHPs on films of hemicellulose, cellulose nanocrystals as well as on gold- and silica surfaces was studied by QCM-D technique^14,27^. In two of our recent studies, we applied cellulose films decorated with ferulate anchor groups for surface polymerization of DHP. The advantage of this procedure is the reproducible dehydrogenative polymerization of lignin monomers^28,29^.

In this work, surface polymerization of coniferyl alcohol on different phenolic anchor groups is carried out. QCM-D measurements provide novel insights into the polymerization speed, deposited amounds, and rigidity of DHPs on thin films. In combination with NMR spectroscopy, this study allows us to conclude structure-property relationships of lignin formation on anchor groups. The observed effects are, among other factors, based on the different radical stabilities of phenolic moieties or benzodioxane formation of caffeate structures.

## Materials and methods

### Materials

Coniferyl alcohol (98%) was received from Alfa Aesar (Karlsruhe, Germany). Horseradish peroxidase (HRP) was purchased from Merck (Darmstadt, Germany). tert-Butyldimethylsilyl chloride was obtained from Apollo Scientific (Stockport, UK). ABTS (diammonium salt of 2,2’-azino-bis(3-ethylbenzothiazoline-6-sulfonic acid)) was purchased from Roche Diagnostics (Mannheim, Germany). Cellulose hydroxycinnamates (1-3) were synthesized by Mitsunobu chemistry^30^. Hydroxybenzoic acid esters of cellulose (4-7) were synthesized according to the literature^31^. DHPs were isolated by alkaline cleavage from the cellulose backbone and extracted with acetone according to the literature^29^. Other chemicals were obtained from VWR (Darmstadt, Germany) or Carl Roth (Karlsruhe, Germany) and were used without further treatment.

Silicon wafers (P/Bor, 100 mm diameter, 525 $\mu$m thickness, <100> orientation) were purchased from Si-Mat (Kaufering, Germany) and broken into 15 mm squares. Quartz crystal microbalance with dissipation monitoring (QCM-D) sensors (QSX301, gold layer) were obtained from Quantum Design (Pfungstadt, Germany).

### Measurements

HSQC NMR spectroscopy was carried out on a Bruker Avance III 600 MHz spectrometer (Bruker, Ettlingen, Germany) with 16 scans and up to 50 mgmL^-1^ sample in acetone-d6/${\textrm{D}_2\textrm{O}}$ (volume ratio of 7:1).

Water contact angles (CAs) were measured with a Dataphysics contact angle measurement system OCA35 (Dataphysics, Filderstadt, Germany) applying the sessile drop method and a drop volume of 3 $\mu$L. Measurements were performed at approximately 3 s from the time of drop deposition. All measurements were conducted at least five times to calculate an average value.

The surface morphology and roughness parameters of the prepared samples were characterised by atomic force microscopy (AFM) in tapping mode with a Keysight 7500 AFM multimode scanning probe microscope (Keysight Technologies, Santa Barbara, CA, USA). The images were taken after drying the samples in a stream of dry high-grade (99.999 wt.%) nitrogen gas. The images were scanned with silicon cantilevers (ATEC-NC-20, Nanosensors, Wetzlar, Germany) with a resonant frequency of 210-490 kHz and a force constant of 12-110 N m^-1^.

QCM-D measurements were carried out with a Q-Sence E4 instrument (Gothenburg, Sweden) at 23.0 °C applying a flow rate of 100 $\mu$L min^-1^. The fundamental frequency of the sensors was $f_0\approx$ 5 MHz, possessing a sensitivity constant of C = 17.7 ng Hz^-1^cm^-2^. According to our previous studies^28,29^ flow experiments were conducted. In brief, the sensors were rinsed with ultrapure water to obtain a stable baseline. After defined timestamps, HRP (1 mg mL^-1^), ${\textrm{H}_2\textrm{O}}$, aqueous solution of 20 mM ${\textrm{H}_2\textrm{O}_{2}}$ and 0.5 mg mL^-1^ coniferyl alcohol were injected. Subsequently, the films were rinsed for 30 min.

For py-GC-MS measurements, an Agilent (Waldbronn, Germany) system (GC 7890 B/MSD 5977) was used. DHPs were decomposed by a Multi-Shot Pyrolyzer EGA/PY-3030D (Frontier Lab, Essen, Germany) at 450 °C. For separation, a ZB-5MS capillary column (30 m$\times$0.25 mm) was heated with 4 K min^-1^ to 50-240 °C. NIST MS Search 2.2 (2014) software allowed the assignment of compounds by comparing spectra in the NIST MS library.

Size-exclusion chromatography (SEC) was conducted with an Azura HPLC/UHPLC unit (Knauer, Berlin, Germany) equipped with an UV detector (254 nm) using dimethyl sulfoxide (DMSO) with 0.1 % ${\textrm{NaNO}_{3}}$ as eluent. For the measurements, sample concentration was adjusted to 1 mg mL^-1^. A set temperature of 60 °C and a flow rate of 0.3  mL min^-1^ was applied at 44 bar. For separation, PolarGel-M (Agilent) and ABOA DMSO-phiL-P-250 (AppliChrom, Oranienburg, Germany) columns were used. Calibration in the range of 180 - 225000 g mol^-1^ was performed with dextran standards. the samples were filtered through a syringe filter (0.45 $\mu$m) with PTFE membrane before injection (30 $\mu$L).

HRP activity was determined by quantification of the oxidation product [ABTS*]^+^ at 405 nm using the UV-Vis spectrometer V-650 (Jasko, Pfungstadt, Germany), as described previously^28^. The values were 251 Umg^-1^ in solution and 650 Um^-2^ on the surface.

### Synthesis of TMS cellulose derivatives, general procedure

A phenolic acid ester of cellulose (3.0 g) was suspended in dry N,N-dimethylacetamide (DMA) (50 mL) and hexamethyldisilazane (HMDS) (31 mL, 150 mmol) was added. Trimethylsilyl chloride (TMSCl) (200 $\mu$L, 1.6 mmol) was added as a catalyst. After stirring the mixture for 1 h at 80°C, it was cooled to room temperature, and isolated by precipitation into deionized water (1.2 L). The obtained material was washed with water and dried in vaccum at 40 °C.

### Gravimetric determination of ${\textrm{SiO}_{2}}$ content and calculation of degree of substitution (DS)

Clean crucibles were heated to 800 °C for 4 h. Subsequently, the samples (ca. 100 mg) were decomposed with oleum (2 mL, 20 % ${\textrm{SO}_{3}}$) at room temperature, and the crucibles were covered overnight. The mixture was solidified via gentle heating with a gas burner. Crucibles were exposed to conditions in the muffle furnace (800 °C, 4 h), when the evolution of fumes stopped.

1 $$\begin{aligned} SiO_{2}[\%]=\frac{DS_{TMS}*M_{SiO_2}*100}{M_{AGU}+DS_{TMS}*M_{S, TMS}+\frac{DS_{TMS}*M_{S, ester}}{R_{DS}}} \end{aligned}$$

$\textrm{M}_{\textrm{AGU}}$ is the molar mass of an anhydroglucose unit. $\textrm{M}_{\textrm{S}}$ is defined as the net molar mass increase caused by the corresponding substituent. $\textrm{R}_\textrm{DS}$ is the ratio between $\textrm{DS}_\textrm{TMS}$ and $\textrm{DS}_\textrm{ester}$ obtained from integral intensities of ^1^H NMR signals of ${\textrm{SiCH}_{3}}$ groups and aromatic CH-protons.

### Surface modifications

Silicon wafers were pretreated for 10 min in an ultrasonic bath with 2-propanol. After rinsing with ultrapure water, wafers were dipped for 1h into piranha solution. Subsequently, wafers were rinsed with ultrapure water and dried in a nitrogen gas flow.

QCM-D sensors and silcon wafers were spin coated at 4000 rpm (acceleration 2500 rpm s^-1^) for 60 s using a POLOS SPIN150i-NPP Single Substrate Spin Processor (Desktop Version) from SPS-Europe B.V. (Putten, Netherlands). Thin films were exposed to hydrochloric acid vapor (from 10 wt.% HCl) in Petri dishes for 15 min at room temperature to cleave TMS groups.

### Synthesis of dehydrogenation polymers (DHPs), general procedure^29^

Phenolic cellulose derivative (1 g) was grinded with an IKA (Staufen im Breisgau, Germany) T 25 digital ULTRA-TURRAX dispersing tool S25N-86 at 15000 rpm in deionized water (10 mL) for 10 min. The dispersion was poured through a fritted filter funnel (G2) and rinsed with ${\textrm{H}_2\textrm{O}}$. Analogous to the QCM-D experiment, HRP (1 mgmL^-1^) was added to the filter cake for 10 min and rinsed with 20 mL ${\textrm{H}_2\textrm{O}}$, subsequently. For lignification, coniferyl alcohol (0.5 mgmL^-1^) in 20 mM ${\textrm{H}_2\textrm{O}_{2}}$ (1 L) was applied. The material was rinsed with 100 mL ${\textrm{H}_2\textrm{O}}$, finally. To isolate the DHPs, the filter cake was treated with NaOH (5 %, 30 mL) for 1 h at room temperature. The clear solution was separated by centrifugation and acidified with diluted HCl (10 %). Formed precipitate was isolated by centrifugation and washed four-times with ${\textrm{H}_2\textrm{O}}$ (40 mL). The material was treated with acetone (40 mL) to dissolve the DHPs. The solid product was obtained by centrifugation evaporation of the clear solution. A typical yield of DHPs obtained with e.g. anchor group **1** (ferulate) was 36 mg.

## Results and discussion

### Silylation of phenolic acid esters of cellulose

For preparation of cellulosic thin films by spin coating, soluble polymers are essential. In our previous work, dissolution of cellulose ferulate in ethyl acetate was achieved via silylation^28^. Trimethylsilyl (TMS) groups can be cleaved off easily after spin coating to obtain cellulosic films possessing phenolic anchor groups.

**Figure 1:**
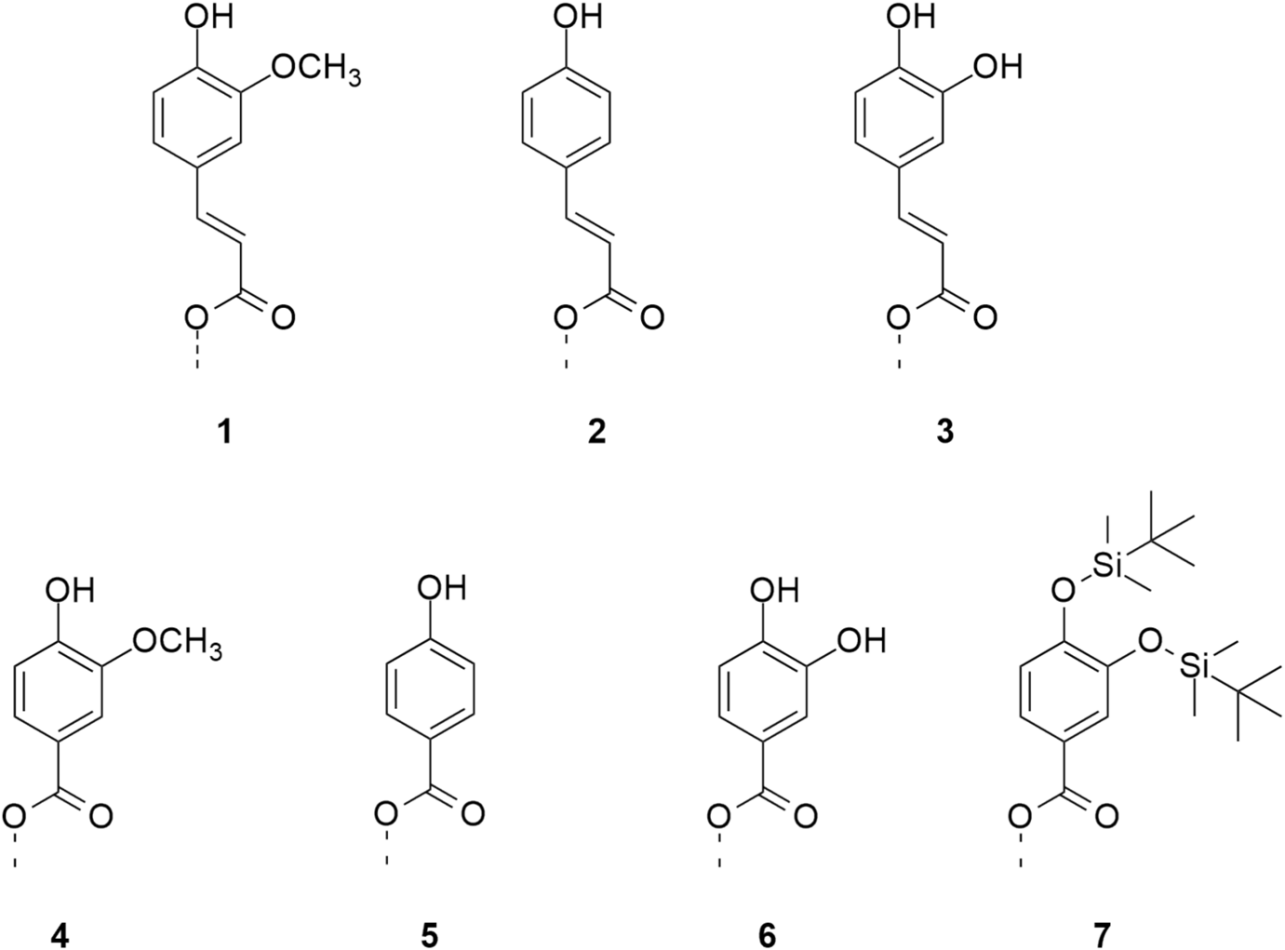
Overview of the phenolic moieties of cellulose esters serving as anchor groups for artificial lignification.

**Figure 2:**
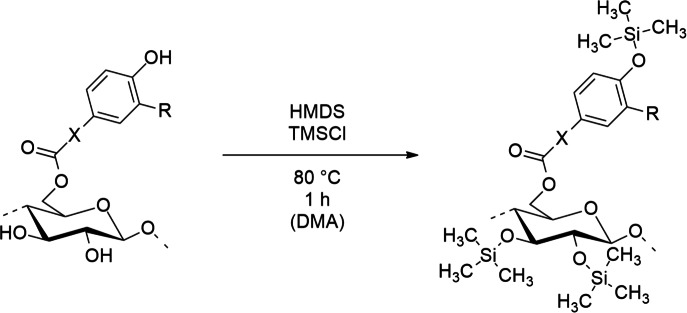
Reaction scheme for silylation of phenolic acid esters of cellulose to enable spin coating of polymers from ethyl acetate.

In this work, cellulose esters with phenolic moieties 1-6 (Fig. 1) were synthesized according to the literature^31^ and subsequently converted with hexamethyldisilazane (HMDS) in the presence of catalytic TMS chloride (Fig. 2). Degree of substitution (DS) values of functional groups were determined by combining NMR spectroscopy and gravimetry. Ratios between TMS- and phenolic groups were determined by integrating the peak intensities of ^1^H NMR spectra. This information was included when calculating the ${\textrm{DS}_\textrm{TMS}}$ from the ${\textrm{SiO}_{2}}$ content, obtained by incineration of decomposed cellulose derivatives. The described method for determination of DS values is more exact than a previous calculation, which was based on the DS of phenolic acid ester (ferulate) prior to silylation^28^. In this way, errors arising from changes in DS during the reaction, by ester cleavage or accumulation in insoluble residues, could be avoided.

As shown in Table 1, $\textrm{DS}_\textrm{TMS}$ is significantly lower for catecholic structures, i.e. caffeate (3, $\textrm{DS}_\textrm{TMS}$ 1.72) and protocatechuate (6, $\textrm{DS}_\textrm{TMS}$ 1.87), than for other phenolic esters ($\textrm{DS}_\textrm{TMS}$ 2.2-2.5). Two adjacent phenolic hydroxyl groups seem to reduce the silylation reaction, which could be caused by intramolecular hydrogen bonds^32,33^. The values are also in line with FTIR spectra showing an increased OH vibration around 3400 to 3500 cm^-1^ compared to monohydroxy phenols 1,2,4, and 5 (Figure S1). However, this difference is not an obstacle for the subsequent application of regenerated cellulosic films.

**Table 1:** DS values of trimethylsilyl phenolic acid esters of cellulose.

Phenolic moiety	No	${\textrm{DS}}_{\textrm{ester}}$ $^{1}$	${\textrm{DS}}_{\textrm{TMS}}$ $^{1}$
Ferulate	1	0.18	2.46
p-Coumarate	2	0.09	2.18
Caffeate	3	0.14	1.72
Vanillate	4	0.21	2.44
p-Hydroxybenzoate	5	0.28	2.53
Protocatechuate	6	0.38	1.87

$^{1}$Degree of substitution determined by combining NMR spectroscopy and gravimetry

### Formation of model films

Cellulosic thin films with phenolic anchor groups (1-6) were obtained by spin-coating from ethyl acetate, followed by removal of silyl groups with HCl vapor. Coating parameters were adapted from literature^28^ to create uniform films for artificial lignification.

AFM images of spin-coated films revealed a uniform surface morphology with tiny holes, which might have arisen from solvent evaporation (Figure S2, A). However, after deprotection, films became smoother without visible flaws (Figure S2, B). The root mean square (rms) roughness of the cellulose ferulate film decreased from 2.8 nm to 1.7 nm.

Water contact angle (CA) measurements indicate similar hydrophobicities (91°-97°) for samples 1-6 arsing from TMS groups (Table 2). The influence of the type and DS value of phenolic moieties seems to be negligible. However, lower $\textrm{DS}_\textrm{TMS}$ value of sample 6 possessing the catecholic pattern lead to less hydrophobicity, i.e. the lowest water CA of 91.5°. FTIR spectra of thin films (Figure S3) show an increased OH signal for caffeate and protocatechuate as already observed for the bulk material (Sect. 3.1).

**Table 2:** Water contact angles [°] of cellulose-based thin films.

Phenolic moiety	No	TMS-protected	Deprotected	DHP@film
Ferulate	1	96.1±1.2	51.0±2.0	46.3±0.6
p-Coumarate	2	97.2±0.9	46.4±1.9	50.3±2.0
Caffeate	3	95.6±1.1	44.0±1.4	51.7±1.5
Vanillate	4	96.6±0.4	38.4±0.5	29.5±1.6
p-Hydroxybenzoate	5	96.8±0.8	41.6±1.1	35.3±0.1
Protocatechuate	6	91.5±0.8	45.1±0.7	61.3±2.3

Successful formation of model films, i.e. complete cleavage of TMS groups, is indicated by disappearance of the Si-C vibration at 1250 cm^-1^ and a pronounced OH signal at 3400 ^-1^. C=O and C=C vibrations of phenolic acid esters of cellulose are still present (Figure S4).

### Lignification on planar surfaces

**Figure 3:**
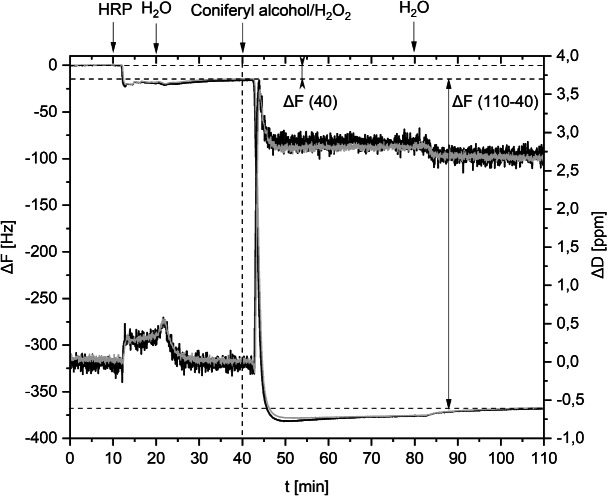
QCM-D online monitoring ($\Delta$F and $\Delta$D values over time) during enzymatic polymerization of coniferyl alcohol, example 6 (DHP@protocatechuate). Double determination is marked black and grey.

Enzymatic polymerization of monolignols on model films in a QCM-D instrument revealed novel insights regarding lignification kinetics. Following our reproducible approach^28,29^, horseradish peroxidase (HRP) was adsorbed on cellulosic model films possessing phenolic anchor groups in the first step (Fig. 3). Loosely bound HRP was rinsed off with water. The frequency shift between two baselines indicated the amount of adsorbed HRP semiquantitatively and was marked as $\Delta$F (40). The experiment continued with introduction of coniferyl alcohol and ${\textrm{H}_2\textrm{O}_{2}}$, which resulted in a remarkable initial slope depending on the reaction speed. The amount of artificial lignin deposited could be evaluated by the difference in $\Delta$F and $\Delta$D at 40 and 110 min.

**Figure 4:**
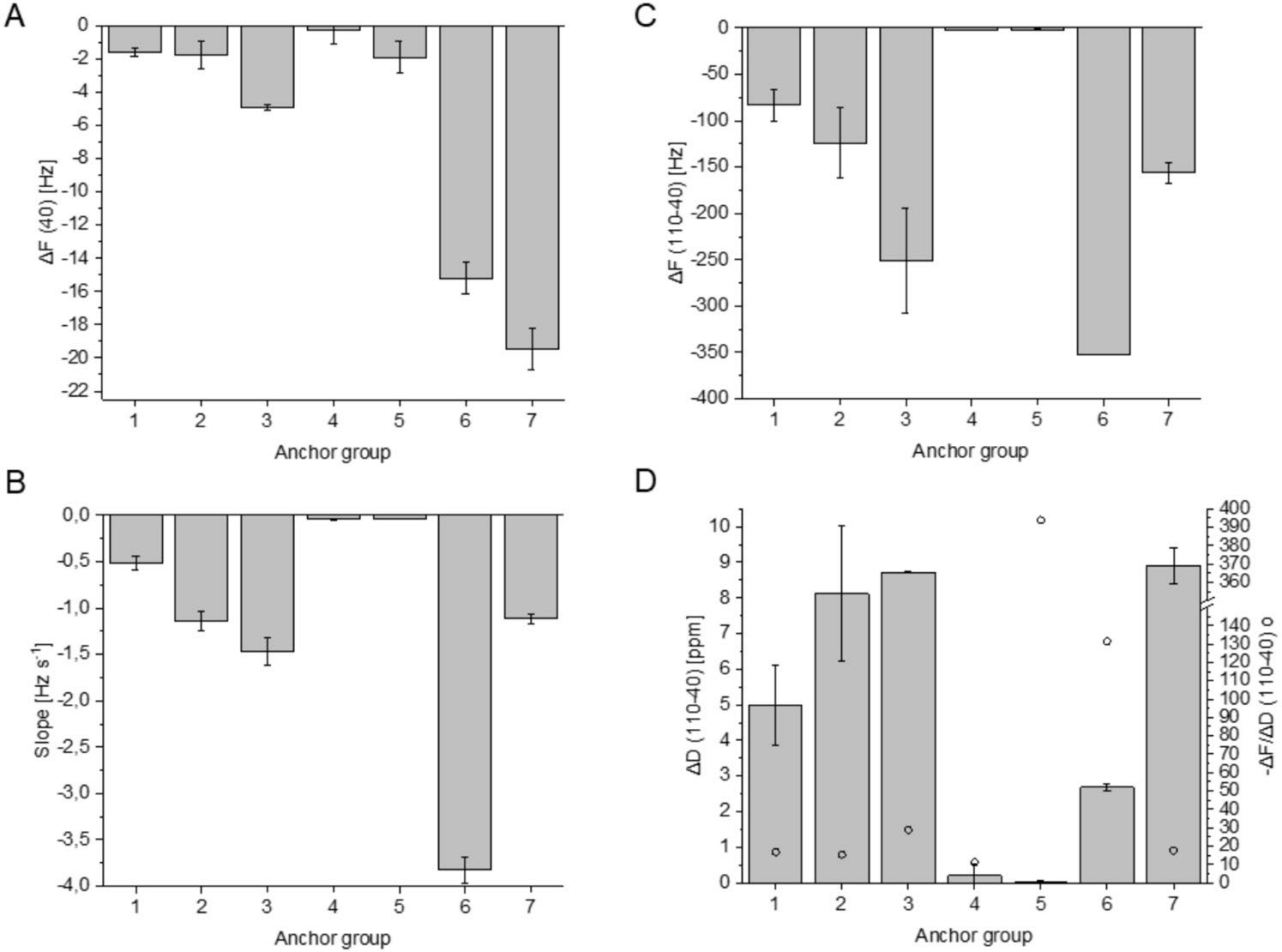
Results of QCM-D experiments dependent on anchor groups 1-7. (**A**): HRP adsorption (semiquantitative), (**B**): Initial slopes indicating velocity, (**C**,**D**): Difference of $\Delta$F and $\Delta$D at 40 and 110 min indicating amount and viscoelastic behaviour of deposited lignin.

Comparing the anchor groups 1-6, there are remarkable differences in HRP adsorption (Fig. 4A). Cellulose protocatechuate (6) performed the strongest adsorption of HRP compared to surfaces 1-5. Protein adsorption is often increased by hydrophobicity of the surface. However, CAs of deprotected films (1-6) show no big differences, which could have explained a pronounced adsorption. Thus, adsorption was not only increased by hydrophobicity, but could be related to catecholic structures (3 and 6) and DS values.

To investigate the effects arising from hydrophobicity, cellulose protocatechuate partially protected with tert-butyl(dimethyl)silyl (TBS) groups (Fig. 2) (7) was used in reference experiments. This very hydrophobic surface (water CA 102°) led to highest HRP adsorption. However, the polymerization speed was not only dependent on the amount of HRP.

The initial slope of the $\Delta$F graph illustrates the speed of mass increase on surface and thus, the polymerization velocity (Fig. 4B). Slope was -3.8 Hz s^-1^ for protocatechuate groups (6), which showed by far the fastest process. Slope of reference 7 (-1.1 Hz s^-1^) is just one quarter of this value, but the amount of adsorbed HRP was higher on this surface (7). This example clearly shows the influence of anchor groups on polymerization.

It should be noted that the surface morphology of film 7 was different from that of samples 1-6. Deep holes with a diameter of up to 500 nm could be observed (Fig. S2, C). There seemed to be distinct wetting issues of the hydrophobic TBS derivative on surface during spin-coating from butanone. It could be assumed that bare silicon or gold was on the ground level of the holes. DHP deposited on film 7 possessed typical particulate structure, but there were empty areas showing size of the holes (Fig. S2, D).

Considering hydroxycinnamates, lignification process accelerated in the order ferulate (1) < p-coumarate (2) < caffeate (3). Vanillate (4) and p-hydroxybenzoate (5) performed only very slow reactions (-0.04 Hz s^-1^). The different behaviour of anchor groups 1 and 2 could be explained by their radical stability, as indicated by redox potential ($\textrm{E}_\textrm{p}$). The values for ferulic acid and its esters are in the range of +400 mV, but $\textrm{E}_\textrm{p}$ is +736 mV for p-coumaric acid^34^. Owing to the low radical stability of p-coumarates, they transfer electrons to other phenols. p-Coumarates accelerate the lignification process in radical transfer reactions, but they are not incorporated integrally into the polymer^35^. In plants, sinapyl alcohol can be dehydrogenated in the presence of catalytic p-coumarate moieties^36–38^. Otherwise, it is a very poor substrate for peroxidases. Moreover, p-coumarate monolignol conjugates are probably controlling the size and 3D structure of lignin^39^.

In the case of caffeate (3), the explanation for a faster lignification is different, since $\textrm{E}_\textrm{p}$ is +370 mV for ethyl ferulate, but +170 mV for ethyl caffeate^34^. High radical stability of the catecholic motif results from a resonance stabilization of the phenoxyl radical intermediate with subsequent ortho-quinone formation. However, catechol moieties are involved in the formation of benzodioxane structues in lignin.

**Figure 5:**
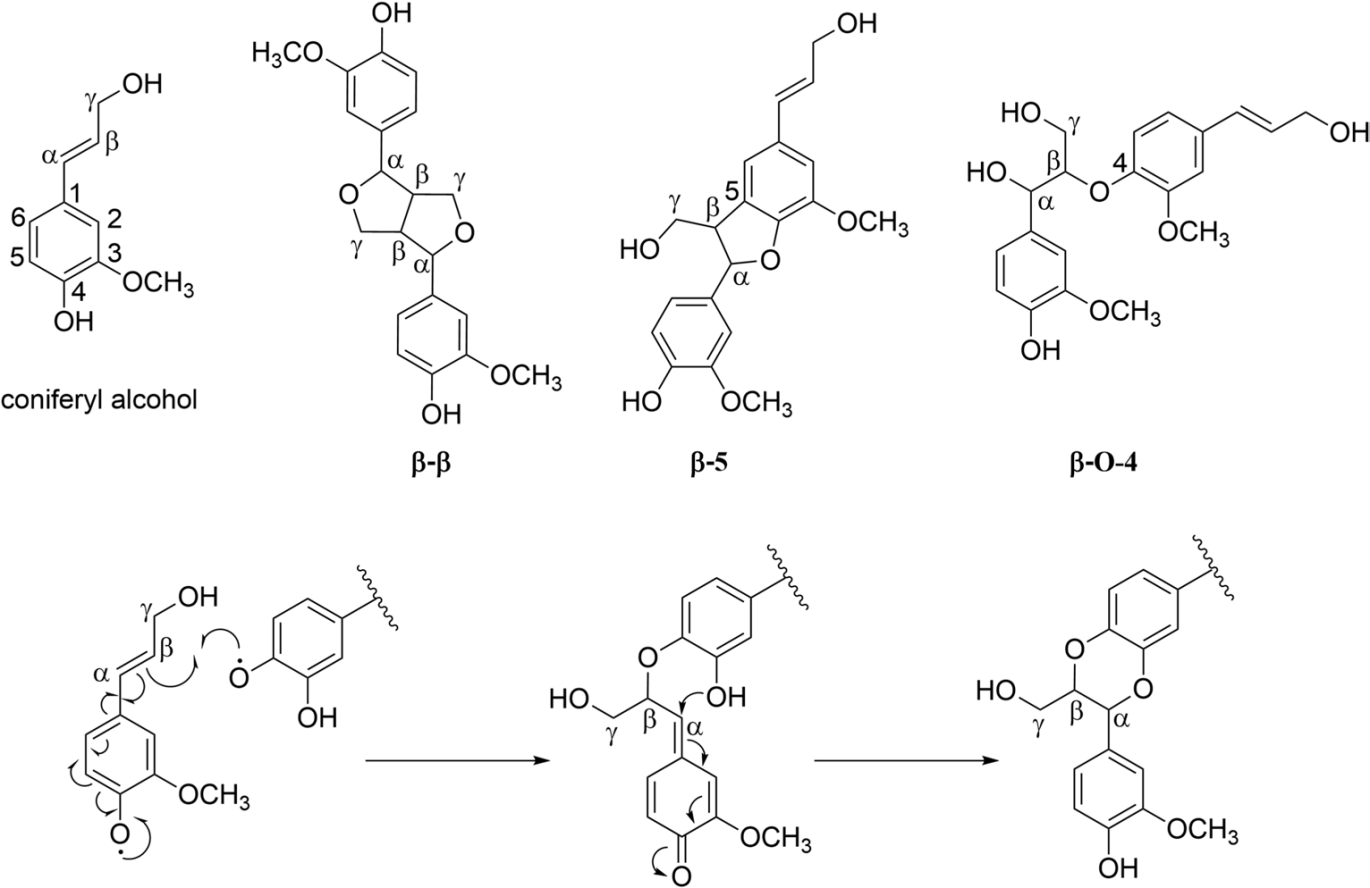
Chemical structures of coniferyl alcohol and its dimers possessing $\beta$-$\beta$, $\beta$-5, and $\beta$-O-4 linkages (top). Reaction mechanism for the benzodioxane formation from coniferyl alcohol and catecholic structures (bottom).

The O-radical reacts with the favored $\beta$-position followed by internal trapping of the quinone methide with the adjacent phenolic OH group to the $\alpha$-position under rearomatization (Fig. 5). This principle was described for the incorporation of 5-hydroxyconiferyl alcohol into lignin of O-methyltransferase-deficient poplars^40^ and Arabidopsis^41,42^. In contrast to conventional $\beta$-O-4 linkages, rotation is blocked, and structures of the macromolecules are less flexible. Moreover, a homopolymer of caffeyl alcohol, so-called C-lignin, was found in vanilla orchid^43^. According to the same mechanism, benzodioxane formation occurs and is kinetically controlled, independent of enzymes or other proteins. This fast trapping step seems to be the reason why caffeate groups (3) show the best lignification performance among the three hydroxycinnamates.

The very high reaction velocity at protocatechuate groups (6) is in line with the electrochemical properties of quinones, which are excellent redox-mediators in biology and electrochemistry^44–46^. Hydroxybenzoates 4 and 5 perform only poor dehydrogenation.

Polymerization velocity also correlated with the final mass deposited on the films. $\Delta$F (110-40) was -83 Hz, -124 Hz, and -251 Hz for 1, 2, and 3 respectively (Fig. 4C). However, there were significant differences in viscoelastic behaviour. DHP@protocatechuate (6) can be considered as very rigid (Fig. 4D, $\Delta$F/$\Delta$D 131) indicating tight bonds between cellulose film and DHP particles. On the other hand, the film of reference 7 was viscoelastic ($\Delta$F/$\Delta$D 17.5), which could be explained by the bulky TBS groups on the film. Thus, formation of covalent bonds between phenolic groups and lignin was more disabled. Anchor groups possessing catecholic structures (3 and 6) were rigid ($\Delta$F/$\Delta$D>25), which suggest an effective covalent bond formation of stiff structures.

Water CA was dependent on the coverage of cellulosic films with lignin particles. On films with vanillate and p-hydroxybenzoate groups, there was almost no deposition of DHPs. These films were hydrophilic, similar to pure cellulose layers (CA 30-35°). On the other hand, high deposition rates could increase the CA up to 61°(Table 2) (6).

### Molecular structure of artificial lignins

To elucidate linkages in artificial lignins formed in the presence of phenolic anchor groups, HSQC NMR measurements were conducted (Figure S5). Synthesis of sufficient amounts of dehydrogenation polymer was carried out by flow through experiments with dispersed material on a suction filter. DHPs were isolated by alkaline cleavage from the cellulose backbone and extracted with acetone^29^.

^1^H and ^13^C NMR resonances were assigned by consideration of HSQC NMR spectra of DHPs from the literature^12^ and data of dimers of coniferyl alcohol (Fig. 5) from the NMR database of lignin and cell wall model compounds^47^. Chemical shifts of position $\alpha$, $\beta$, and $\gamma$ could be assigned to the linkage types $\beta$-$\beta$, $\beta$-5, and $\beta$-O-4 as well as the $\beta$-O-4 benzodioxane motive.

Cross-peaks at 5.5/88 ppm and 4.8/73 ppm arising from position $\alpha$ of the coniferyl units were integrated to determine the percentage of $\beta$-5 and $\beta$-O-4 linkages. For DHP formed at cellulose caffeate, the benzodioxane motif could be distinguished at 4.9/77 ppm. $\beta$-$\beta$ bonds were quantified using the signal of position $\beta$ at 3.0/54 ppm.

All DHP samples possessed approximately 50 % $\beta$-$\beta$ linkages (Table 3). Ferulate (1) and coumarate (2) moieties lead to similar percentages of $\beta$-5 ($\approx$30 %) and $\beta$-O-4 (16 %) bonds. Lignification on cellulose caffeate (3) yielded DHP with low $\beta$-5 content (22 %) and many $\beta$-O-4 linkages. As assumed during QCM-D experiments (Sect. 3.3), the catechol structure was incorporated into lignin by the formation of the benzodioxane motif, which constitutes 9 % of the 32 % $\beta$-O-4 bonds. Protocatechuate (6) performed as an excellent redox mediator, but no benzodioxane structure could be observed. However, $\beta$-O-4 content was high (24 %). No DHP could be isolated from cellulose vanillate or without a matrix. DHP formed with p-hydroxybenzoate (5) consists of high amounts of $\beta$-5 linkages (40 %) and fewer $\beta$-O-4 bonds (8 %).

In general, DHPs obtained by our flow through experiments showed typical structures of Zulauf (ZL) lignin. The content of $\beta$-$\beta$ linkages was very high, but 5-5 and $\beta$-1 could not be observed in the NMR spectra. This could be explained by high concentration of monomer radicals leading to a pronounced monomer-monomer coupling and less oligomer-oligomer coupling^48^.

In context with the use of DHPs as synthetic model of lignin, a similar chemical structure compared to native lignin is aimed^12^. For example, DHPs obtained from coniferyl alcohol possess higher $\beta$-5 and $\beta$-$\beta$ contents but lower amount of $\beta$-O-4 bonds than natural lignin. Thus, a pronounced increase of $\beta$-O-4 linkages is desired and could be achieved by phenolic groups 3 and 6. The anchor group caffeate increased the $\beta$-O-4 content by the formation of benzodioxane structures. Moreover, high deposition rates provide hydrophobic DHP aggregates, which should be beneficial for $\beta$-O-4 bonding^12^. In particular, this could be the effect of protocatechuate groups, which did not lead to benzodioxane structures but rather high $\beta$-O-4 content. On the other hand, anchor groups with poor performance lead to very low $\beta$-O-4 content (5) or even no aggregation of DHP (4).

**Table 3:** Characterization of dehydrogenation polymers (DHPs) formed at dispersed phenolic cellulose derivatives.

Sample No	Molecular weight distribution$^{1}$			Linkage type [%]$^{2}$		
	$\textrm{M}_\textrm{n}$ [g mol^-1^]	$\textrm{M}_\textrm{w}$ [g mol^-1^]	PDI	$\beta$-$\beta$	$\beta$-5	$\beta$-O-4
DHP@ferulate(1)	682	1550	2.27	56	28	16
DHP@coumarate(2)	915	5501	6.01	52	31	16
DHP@caffeate(3)	1070	6902	6.45	46	22	32^3^
DHP@hydroxybenzoate(5)	891	1741	1.95	52	40	8
DHP@protocatechuate(6)	540	1119	2.14	54	22	24

$^{1}$Determined by size exclusion chromatography.

$^{2}$ Determined by HSQC NMR spectroscopy.

$^{3}$ Contains 9 % of benzodioxane structure

Py-GC-MS chromatograms (Figure S6) of DHPs show specific peaks arising from the anchor groups incorporated into the lignin structure (Figure S7). The signals of ferulic acid, p-coumaric acid, and p-hydroxybenzoic acid are clearly visible. For the caffeate structure, the fragments catechol, methyl catechol, and ethyl catechol are visible. Catechol and methyl catechol were also detected during pyrolysis of protocatechuate moities. Moreover, pyrolysis of p-coumaric acid leads to an intense signal of coumaran. It could be assumed, that p-coumaric moieties are less covalently linked to lignin than e.g. ferulic acid, which is comparable to the situation in natural plants (Sect. 3.3).

The molecular mass distribution of DHPs obtained with hydroxycinnamates correlates with the reaction velocities and the deposited amounts observed in QCM-D experiments (1<2<3) from ferulate ($\textrm{M}_\textrm{w}$ 1550) to p-coumarate ($\textrm{M}_\textrm{w}$ 5501) to caffeate ($\textrm{M}_\textrm{w}$ 6902, Table 3). However, molar masses of DHPs from hydroxybenzoates (5-6) are lower in general, and there seems to be no correlation with the lignification speed or deposited mass.

## Conclusions

The synthesis of soluble phenolic acid esters of cellulose enabled spin coating of biopolymers possessing different anchor groups. This methodology allowed reproducible lignification studies on thin films by means of QCM-D. On the one hand, the velocity of dehydrogenation polymerization of coniferyl alcohol could be estimated from the slopes of the curves. On the other, frequency shifts and dissipation values allowed conclusions about the rigidity of artificial lignin and its attachment to cellulose films. HSQC NMR spectroscopy was used to quantify the linkage types $\beta$-$\beta$, $\beta$-5, and $\beta$-O-4 including the benzodioxane structure of DHP obtained in a preparative flow through experiment.

Comparing the hydroxycinnamates, dehydrogenation polymerization was accelerated and the deposited amount was increased in the order ferulate < p-coumarate < caffeate. As observed in plants, p-coumarate accelerated dehydrogenation polymerization via catalytic radical transfer to other phenolic moieties. This could be explained by its high redox potential, i.e. poor radical stability. The improved lignification by caffeate was related to the formation of benzodioxane structures. Owing to the well mediating redox properties of quinones, protocatechuate anchor groups perform excellently the reaction, whereas vanillate and p-hydroxybenzoate lead to almost no deposition of lignin. Catecholic moieties were the most active anchor groups of our study leading to an increased $\beta$-O-4 content, which is interesting for modeling real plant lignin.

The formation of the benzodioxane motif is known from lignin in O-methyltransferase-deficient plants. This linkage is more stable than an ordinary $\beta$-O-4 bond and complicates pulping^48^. On the other hand, the trapping reaction with catechols prevents cross-linking with other cell wall components, e.g. polysaccharides, and improves digestability^49^. Owing to rigidity and linearity of these lignins, a series of useful features are provided. One example is the synthesis of carbon fibers with high performance. Carbonization of poly-(caffeyl alcohol), which is known as C-lignin, compares very well to commercial materials from polyacrylonitrile^50^.

This approach allowed us to study the structure-property relationships in lignification on natural and non-natural phenolic anchor groups without considering genetic information. It provides a useful puzzle piece in the design of lignin structures to achieve advancements in pulping, biofuels, agriculture, and functional materials.

## Supplementary Information

Supplementary Information.

## Data Availability

Data are available in the Supplementary Information (SI) and on reasonable request from the corresponding author.
