# Supplementary material for: Effect of phenolic anchor groups on enzymatic polymerization of coniferyl alcohol at cellulosic interfaces
Source: Sci Rep. 2025 Sep 12;15:32471. doi: 10.1038/s41598-025-18530-9 (PMC12432180; doi:10.1038/s41598-025-18530-9)
Supplement: Supplementary file 1 — Supplementary Information. [file 41598_2025_18530_MOESM1_ESM.pdf]

Supplementary information

Effect of phenolic anchor groups on enzymatic polymerization of coniferyl alcohol at cellulosic interfaces

Thomas Elschner[1]*, Jakob Schönrich[1], Matej Bračič[2], Tina Maver[3], Uroš Maver[3], Steffen Fischer[1]

[1] Institute of Plant and Wood Chemistry, Dresden University of Technology, Pienner Str. 19, Tharandt, 01737, Saxony, Germany.*

[2] Faculty of Mechanical Engineering, Institute of Engineering Materials and Design, University of Maribor, Smetanova 17, Maribor, 2000, Slovenia.

[3] Faculty of Medicine, Department of Pharmacology, University of Maribor, Taborska ulica 8, Maribor, 2000, Slovenia.

Email: thomas.elschner@tu-dresden.de

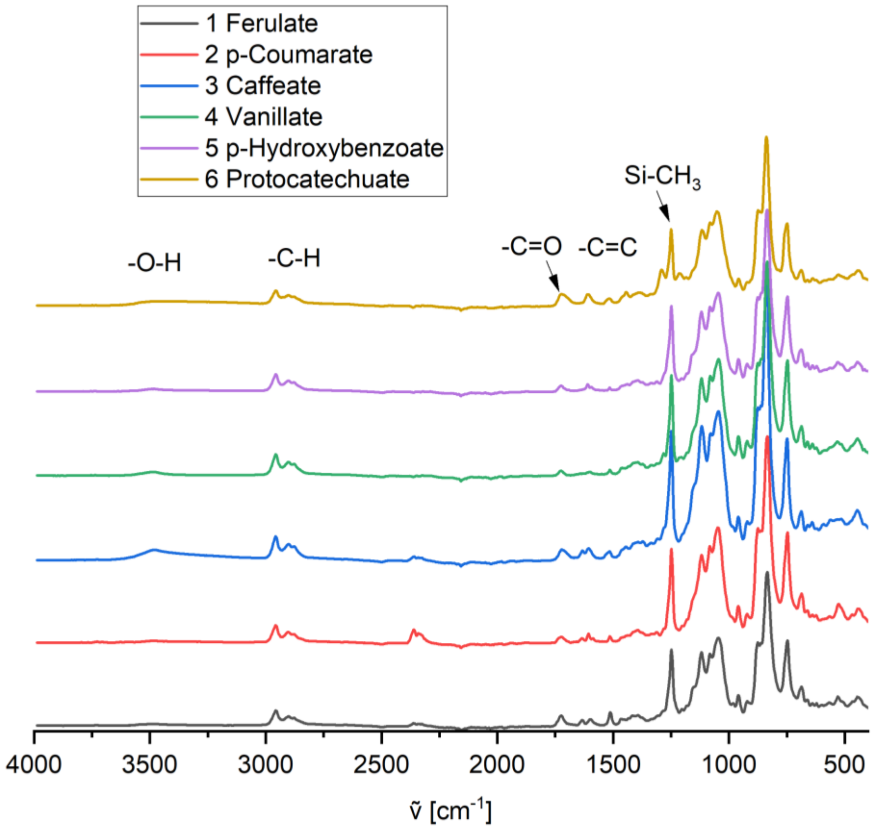

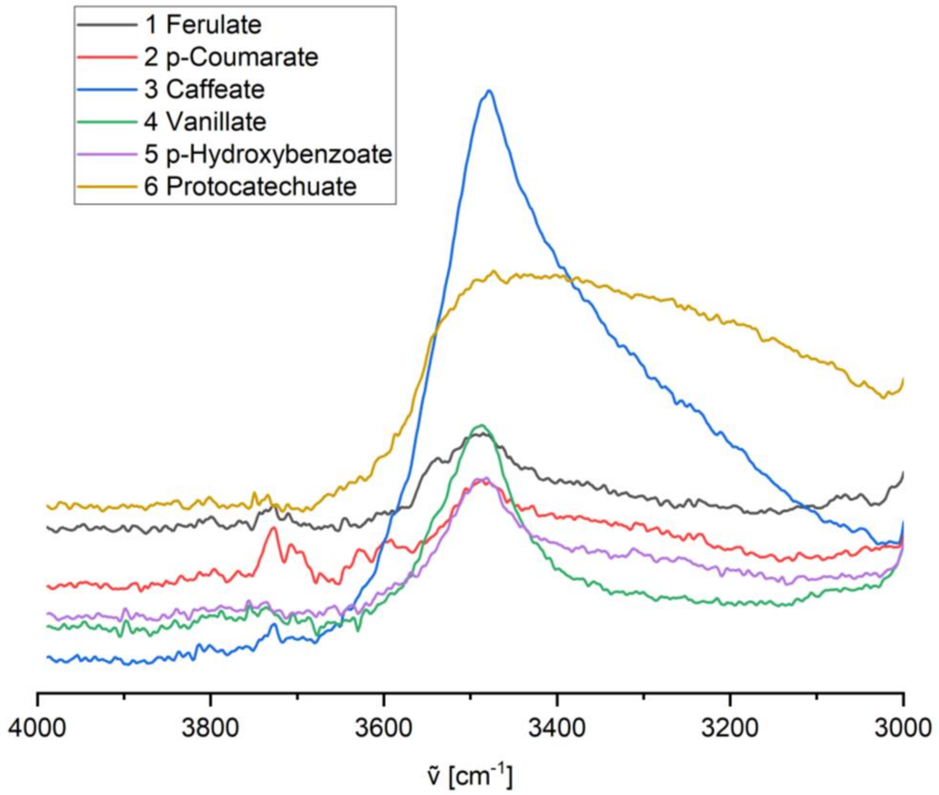

Figure S1: FTIR spectra of silylated (TMS) phenolic acid esters of cellulose.

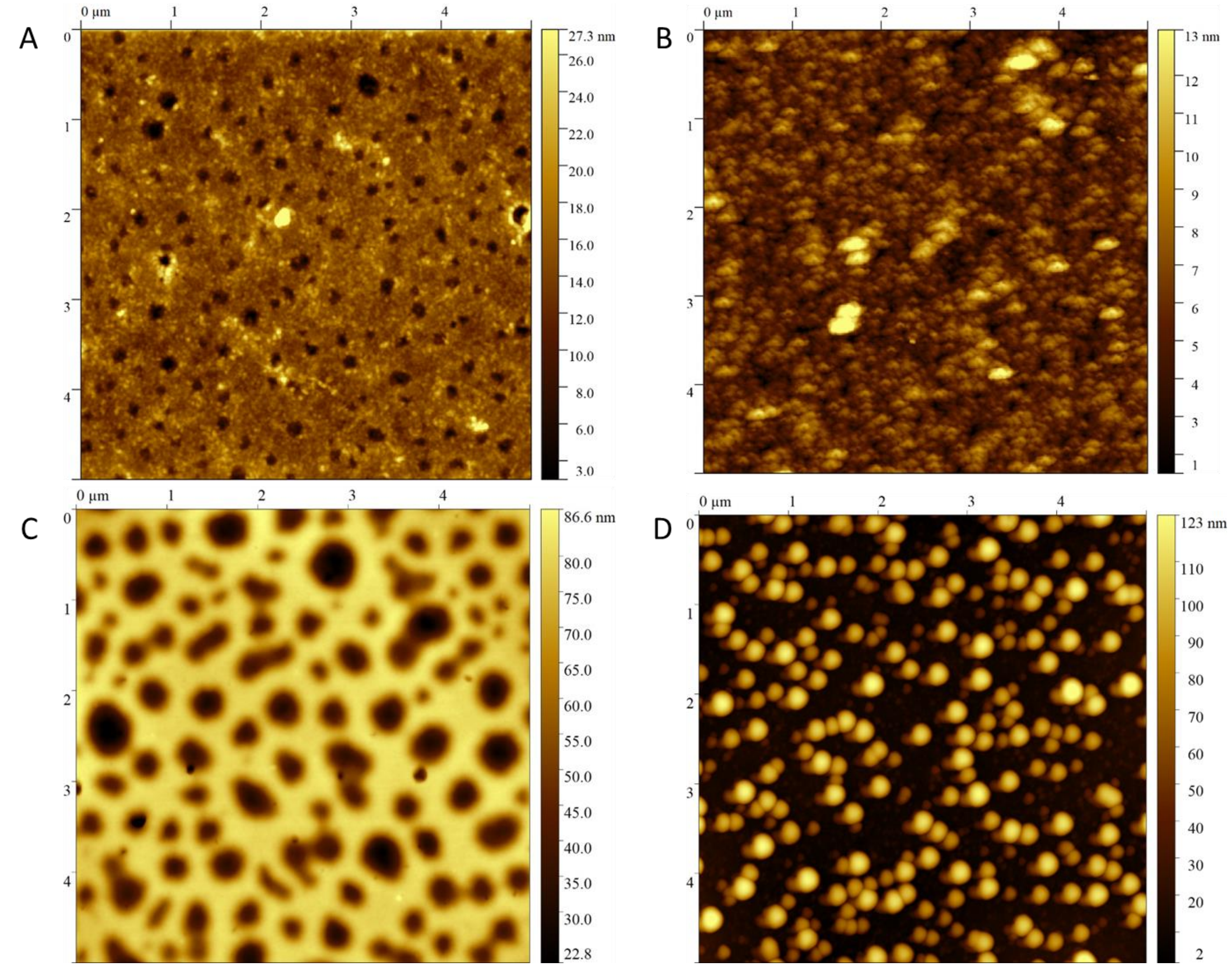

Figure S2: AFM images (5 µm × 5 µm):

A) TMS cellulose ferulate(1) film (RMS 2.847 nm)
B) Cellulose ferulate(1) film (RMS 1.692 nm)
C) Cellulose TBS-protocatechuate(7) film (RMS 14.29 nm)
D) DHP@7 film (RMS 31.73 nm)

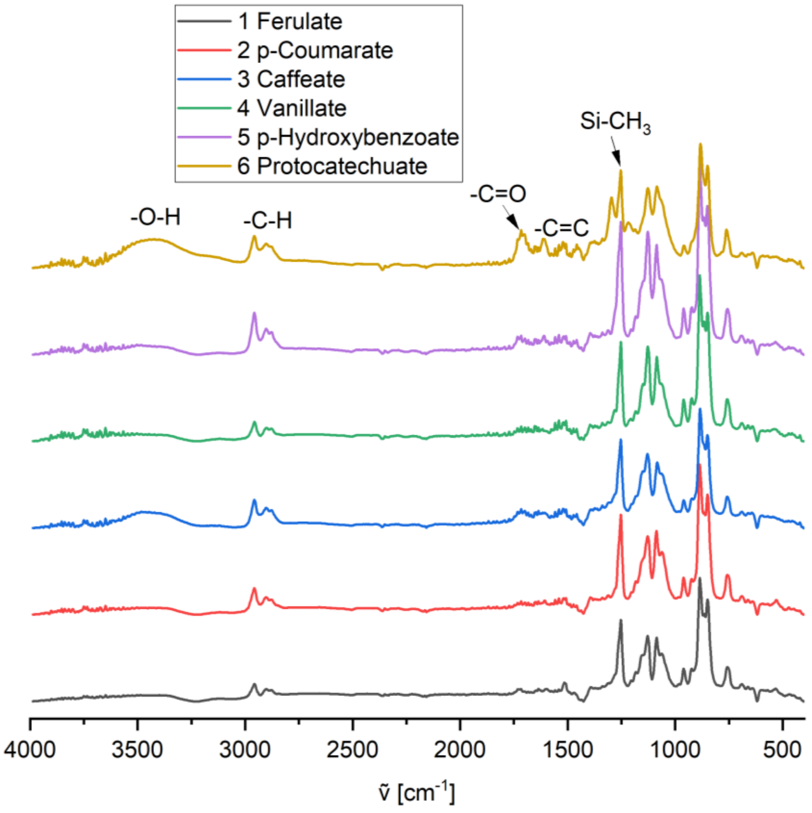

Figure S3: FTIR spectra of thin films obtained by spin coating.

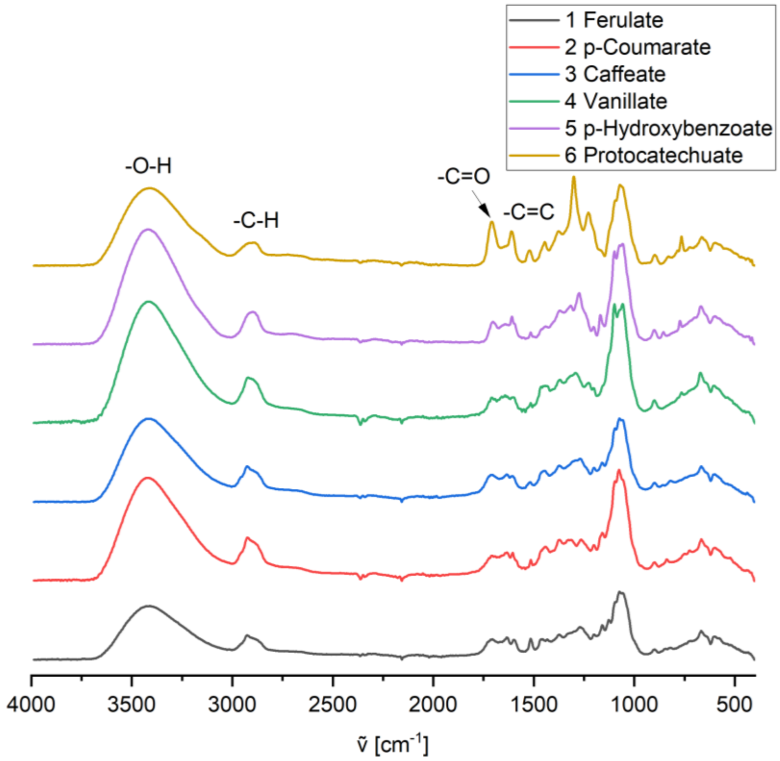

Figure S4: FTIR spectra of thin films after treatment with HCl vapor (TMS groups removed).

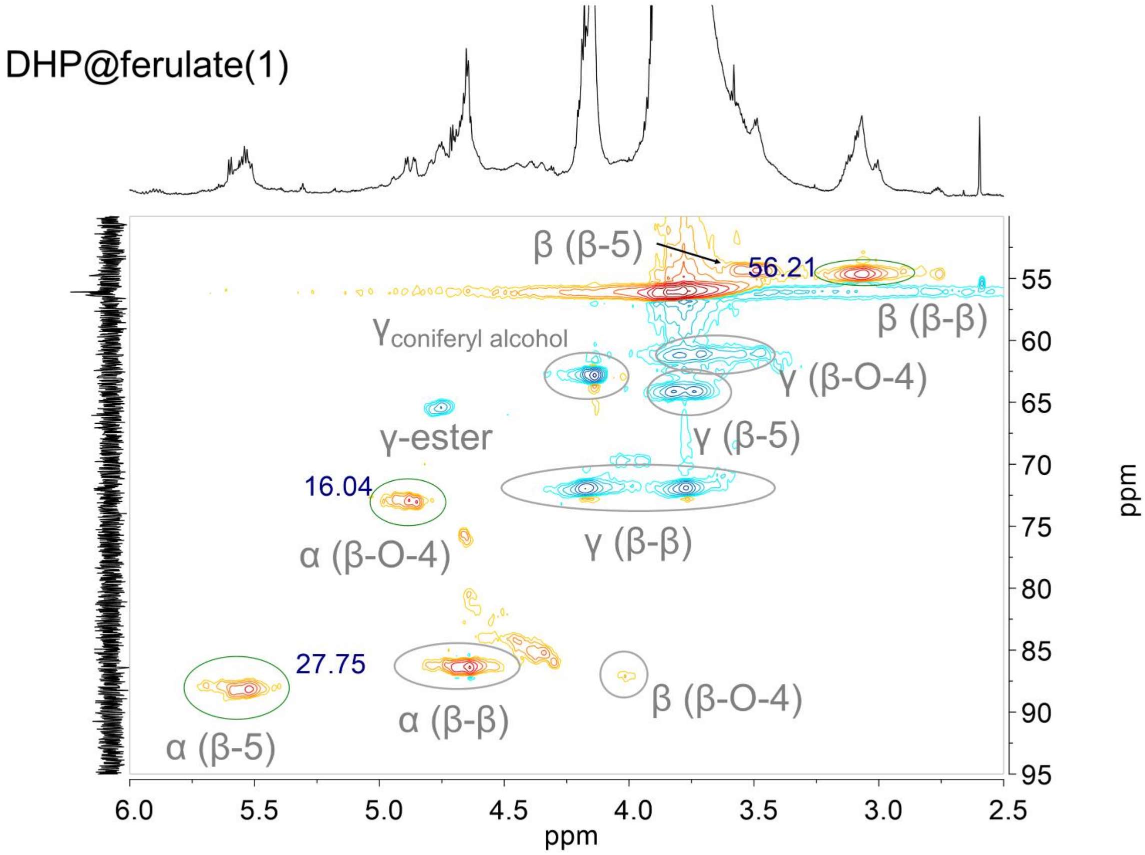
DHP@ferulate(1)
β (β-5)
56.21
β (β-β)
γconiferyl alcohol
γ (β-O-4)
γ (β-5)
γ-ester
16.04
α (β-O-4)
γ (β-β)
27.75
α (β-β)
β (β-O-4)
α (β-5)
ppm
ppm

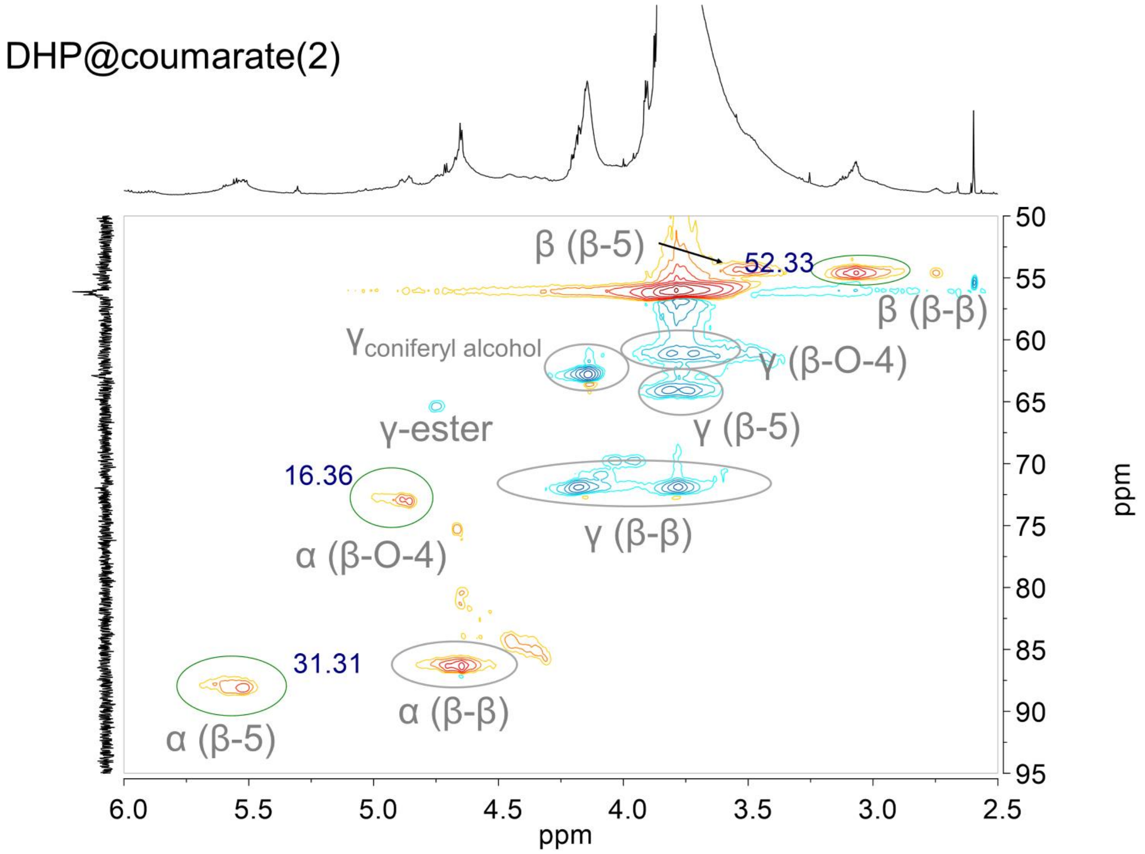
DHP@coumarate(2)
β (β-5)
52.33
β (β-β)
γconiferyl alcohol
γ (β-O-4)
γ (β-5)
γ-ester
16.36
α (β-O-4)
γ (β-β)
31.31
α (β-β)
α (β-5)
ppm
ppm

DHP@caffeate(3)

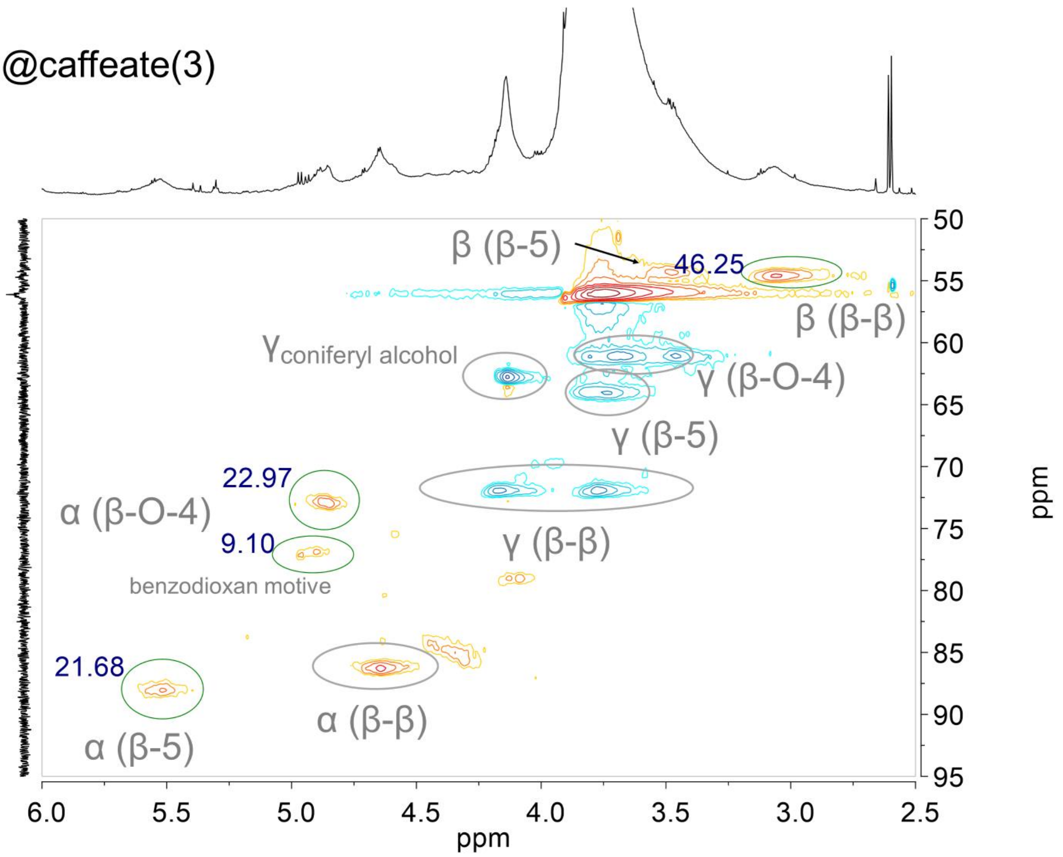

DHP@hydroxybenzoate(5)

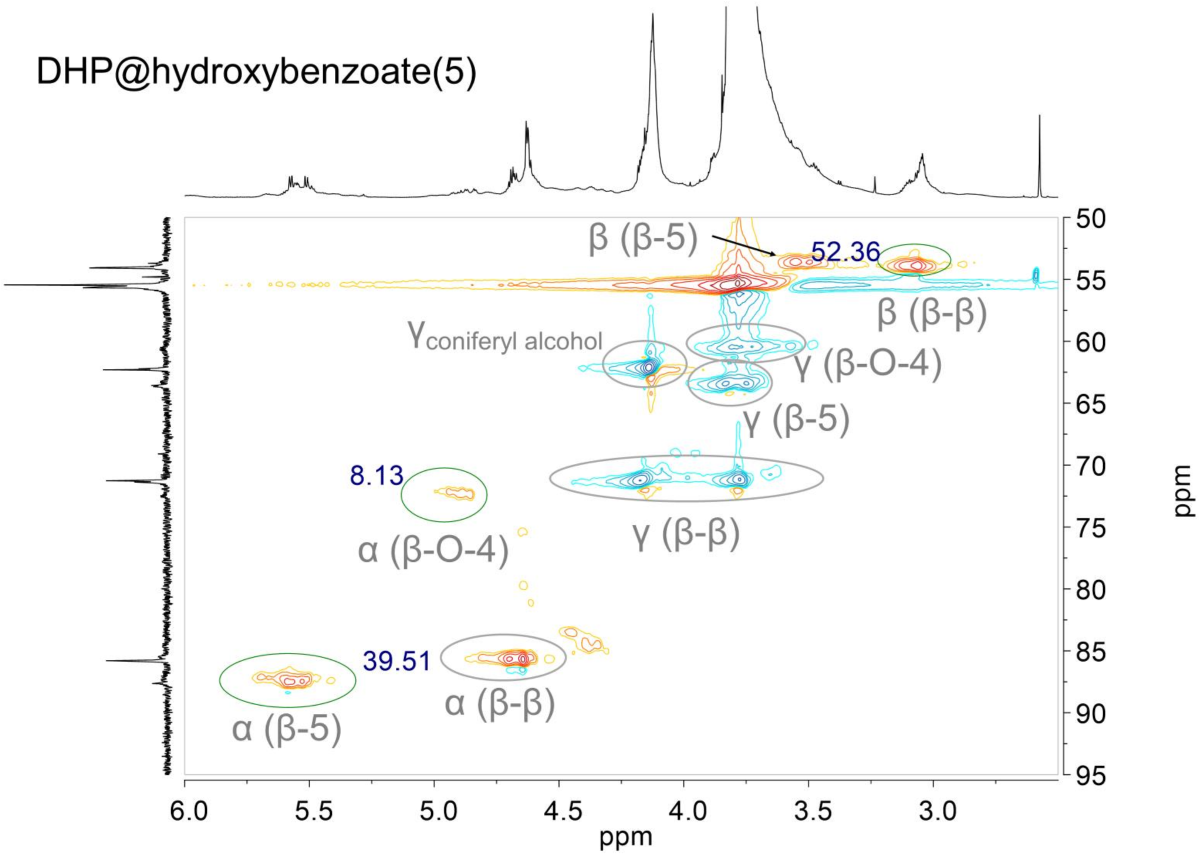

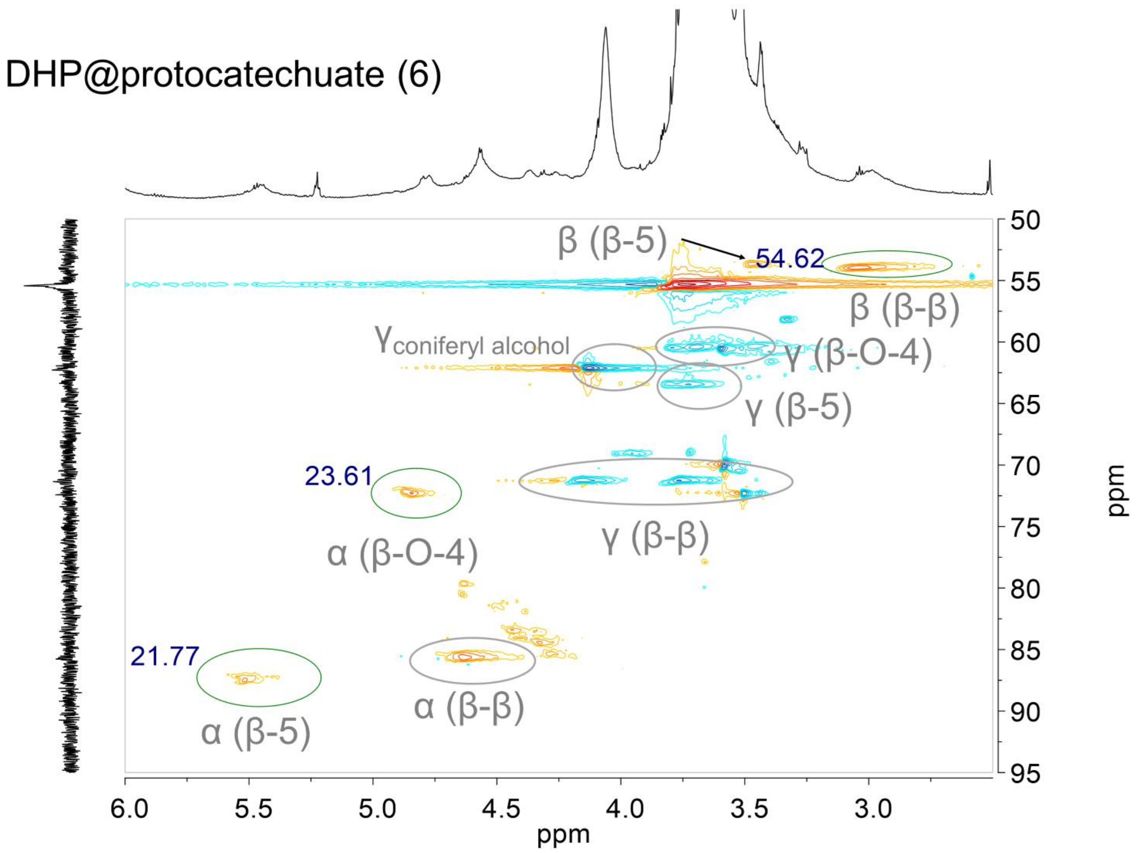

Figure S5: HSQC NMR spectra of DHP recorded in acetone-d6/D_2O.

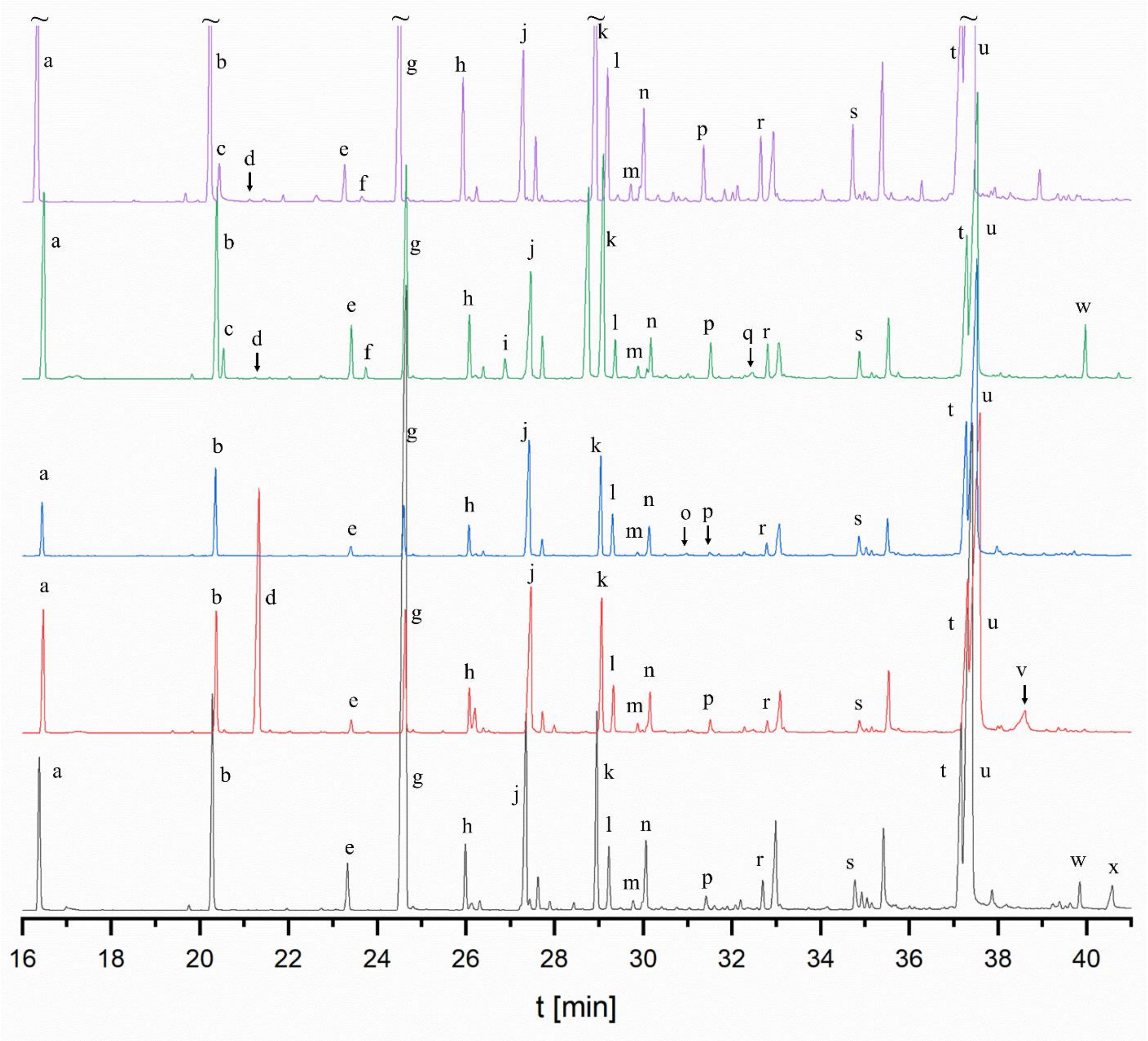

Figure S6: Chromatograms of pyrolysis products from DHP@anchor groups: protocatechuate (purple), caffeate (green), p-hydroxybenzoate (blue), p-coumarate (red), and ferulate (black) obtained by Py-GC-MS measurements.

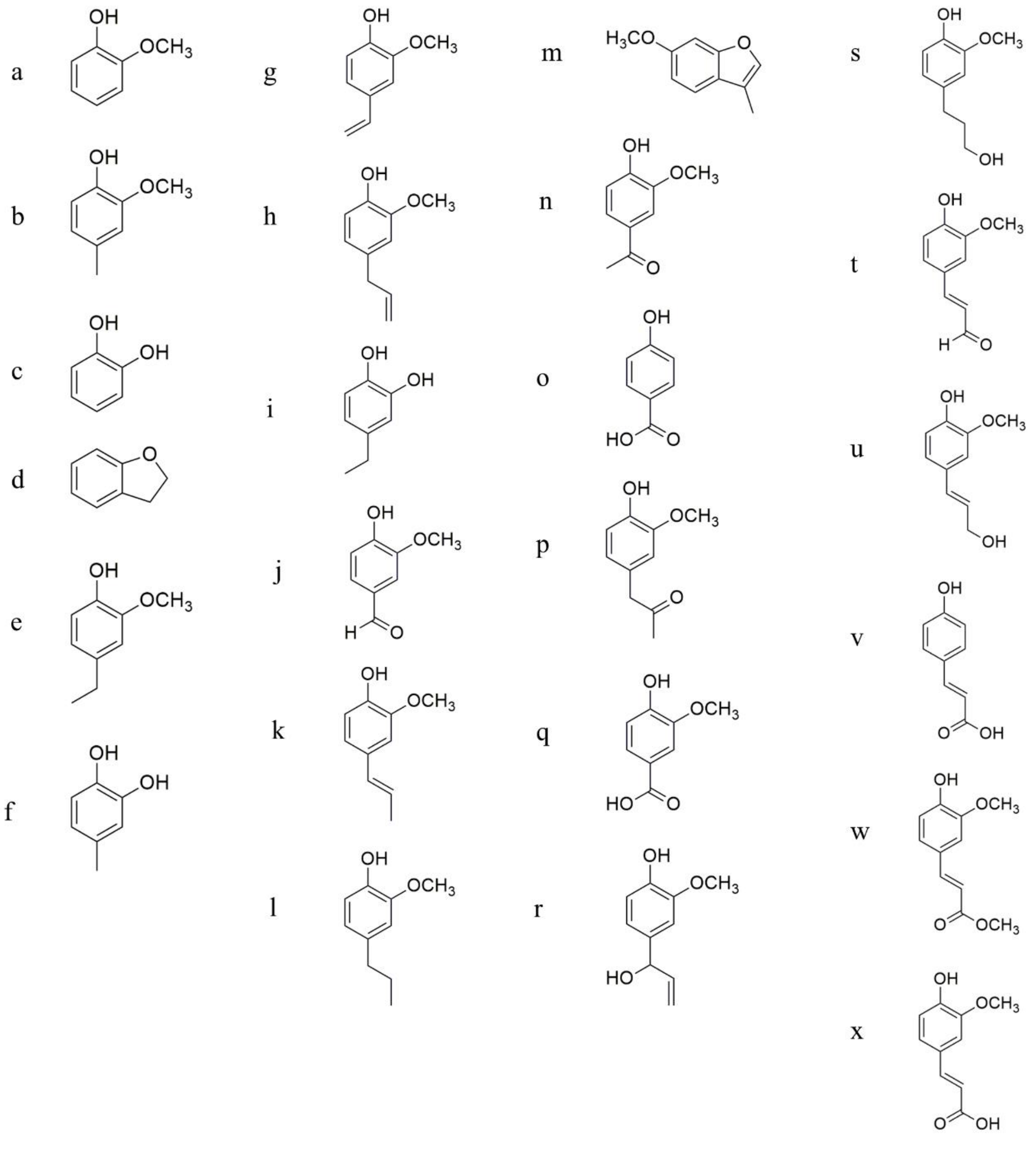

Figure S7: Chemical structures assigned to chromatograms in Figure S6.
